# Pennsylvania State Veterinary Medical Association

**Published:** 1896-09

**Authors:** 


### PENNSYLVANIA STATE VETERINARY MEDICAL ASSOCIATION.

Report of Committee on Resolutions at the annual meeting in March:

We, your committee, have had placed before us the Army Bill as at present pending before Congress. We believe that this measure is a much-needed one, and we most heartily recommend that this Association indorse this measure, and urge upon its members the most earnest support to insure its passage.

Adopted.

WHEREAS, At the last session of the State Legislature said body created the Department of Agriculture, that the work falling in this line should be carried on more efficiently and effectively, and be it

*Resolved*, That we heartily concur in the creation of this department, because we believe much more valuable service can thus be rendered through well-established departments, with heads, where responsibility of the work may be better fixed, and where there may be hearty co-operation of the several lines of work now placed under the control of this department, and which are so closely allied as to lose much of their value when under widely scattered bureaus or boards.

Carried.

WHEREAS, A number of diseases afflict the domestic animals of this State that have never received the careful scientific study that is necessary to discover their causes and the methods to be employed in preventing and treating them, and especially so-called cerebro-spinal meningitis of horses, osteoporosis, abortion of cattle, the diseases of fowls, etc.

WHEREAS, Many of the questions involved in a study of these diseases can only be solved by co-operation between the practitioners who are brought in contact with them and an investigator who has superior facilities for research; and

WHEREAS, It is of great importance to the public and the live-stock industry of this State that the above-mentioned and other diseases should be carefully studied, be it

*Resolved*, That the State Live-stock Sanitary Board is hereby requested to direct the State Veterinarian to co-operate with veterinarians and others of this State who have had experience with these very imperfectly understood diseases, and arrange to conduct investigations with a view of discovering their causes and the means to be employed in preventing and curing of them; and be it further

*Resolved*, That a copy of these resolutions be forwarded to the President of the Live-stock Sanitary Board.

Carried.

WHEREAS, It has pleased Almighty God in His wise dispensation to remove from our midst our late member, DR. W. U. CUSTER, be it

*Resolved*, That while we bow humbly to His will, we feel keenly the loss that has fallen to our lot, for in our late member we had one devoted to his calling, an earnest member of this organization, and a man honored and respected by the whole community in which he resided; and further be it

*Resolved*, That a copy of these resolutions be spread upon the minutes of our Association, a copy sent to the family of the deceased and to the several veterinary journals.

Carried. Respectfully submitted,

W. HORACE HOSKINS,
Chairman,
THOMAS B. RAYNER,
OTTO NOACK,
Committee.

F. S. Allen, "Acetanilide;" H. B. Felton, "Canine Distemper;" S. J. J. Harger, "Glanders;" John R. Hart, "Azoturea;" W. H. Hoskins, "Pyoktanin;" J. C. Michener, "Parturient Apoplexy;" Charles Williams, "Cannabis Indica;" Jacob Helmer, "Homœopathy *vs.* Regular Treatment in Veterinary Medicine;" James T. Ross, "Management of Cases of Parturient Apoplexy Homœopathically;" and M. E. Conard, "Abortion," will be the speakers on the topics named at the Pennsylvania State Veterinary Medical Association's semi-annual meeting at Reading on Tuesday, October 6, 1896. The following committees will report through their respective chairmen: Leonard Pearson, Sanitary Science and Police; John R. Hart, Legislative Committee; W. L. Zuill, Intelligence and Education.

